# The National Insurance Bill

**Published:** 1911-08-12

**Authors:** Richard J. Coles

**Affiliations:** Secretary-Superintendent, Addenbrooke's Hospital, Cambridge.


### INSTITUTIONAL LETTER-BOX.

### THE NATIONAL INSURANCE BILL.

*To the Editor of* The Hospital.

Sir,—With reference to your Editorial comments upon my letter to you in last week's issue of The Hospital, it may interest you to know that I have thoroughly studied the Bill as well as the amendments advocated in The Hospital, and I do not hesitate to say that the latter are as dangerous to the permanent preservation of the voluntary system as the Bill is designed towards its destruction. If we are earnest in our desire to preserve the voluntary hospitals, the only plan is to reject State aid in any form and at all costs. I have had experience of State-aided institutions.

I am, Sir, yours faithfully,
Richard J. Coles, Secretary-Superintendent,
Addenbrooke's Hospital, Cambridge.

